# A new pathway for pyrite formation in low-sulfate sediments driven by mineralization of reduced organic sulfur

**DOI:** 10.1016/j.fmre.2023.08.003

**Published:** 2023-09-14

**Authors:** Chenhui Wei, Shujun Yin, Andreas Kappler, Shu Tao, Dongqiang Zhu

**Affiliations:** a School of Urban and Environmental Sciences, Key Laboratory of the Ministry of Education for Earth Surface Processes, Peking University, Beijing 100871, China; b Geomicrobiology, Department of Geoscience, University of Tuebingen, Tuebingen 72076, Germany; c Cluster of Excellence: EXC 2124: Controlling Microbes to Fight Infection, Tuebingen 72076, Germany

**Keywords:** Pyrite, Sulfide, Low-sulfate sediments, Reduced organic sulfur, Mineralization

## Abstract

Although pyrite is the main sedimentary form of sulfur, an ample mechanistic comprehension of its formation in low-sulfate environments is lacking. Applying high depth-resolution multigeochemical and stable sulfur isotope composition (δ^34^S) analysis of a sediment core recovered from a large shallow freshwater lake (Baiyangdian) in north China, we show that the pyrite forms dominantly in the top 4 cm layer and the participating sulfide stems primarily from mineralization of reduced organic sulfur in biomass. This mechanism was further verified by the formation of pyrite in anoxic incubation of biomass (*Ceratophyllum demersum* L. or *Spirulina*) with hematite in the absence of external sulfate. This finding reveals an alternative pathway other than microbial sulfate reduction (MSR) for producing sulfide to form pyrite in low-sulfate sediments.

## Graphical abstract

**Figure:**
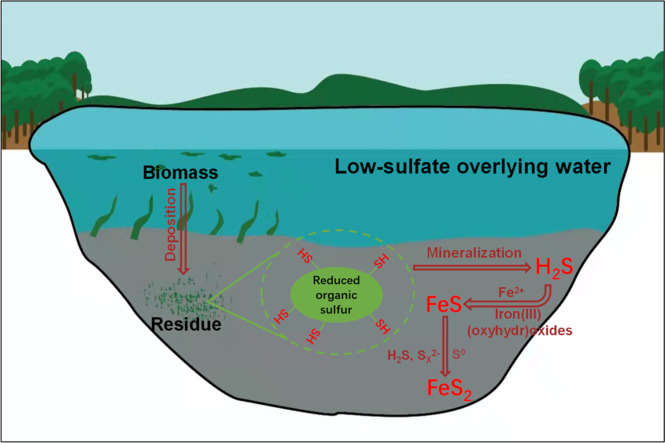

## 1 Introduction

The formation and burial of authigenic pyrite (FeS_2_) in sediments has been recognized as the dominant pathway for the permanent immobilization of sulfur [1]. It also strongly affects the speciation and fate of many toxic trace metals (e.g., mercury and cadmium) that react with sulfide to form stable precipitates [2]. Moreover, the features (e.g., isotope compositions) of sedimentary FeS_2_ can provide important information on the evolution of the Earth [3], [4]. In anoxic sediments, sulfide (HS^−^) can react with ferrous iron ion (Fe^2+^) produced from microbial iron(III) reduction (MIR) or directly with iron(III) (oxyhydr)oxides to form mackinawite (FeS_m_) and/or iron sulfides (FeS), which subsequently react with sulfide or polysulfides to form the more stable FeS_2_ [5], [6], [7]. As a prerequisite for FeS_2_ formation, sulfide in natural sediments is proposed to be mainly generated by microbial sulfate reduction (MSR) coupled to organic matter mineralization [1]. MSR is often the dominant sulfide-producing pathway in marine sediments as the abundant sulfate (∼28 mM) in the overlying seawater can readily diffuse and drive anaerobic carbon mineralization [1]. However, the importance of MSR for producing sulfide in freshwater sediments remains controversial because of the typically much lower sulfate concentrations (generally less than 500 µM).

Plant and algae materials deposited on the sediment surface contain appreciable contents of organic sulfur (0.3% to 2% of dry weight) which is composed of reduced organic sulfur (∼40%, mainly thiols/R-SH and thioethers/R-S-R) and oxidized organic sulfur (∼60%, mainly ester sulfates/R-O-SO_3_H) [8], [9], [10], [11], [12]. The organic sulfur in the sedimentary biomass is mostly mineralized to inorganic sulfur in the subsurface layer, including sulfide, sulfite and sulfate depending on the chemical valence of organic sulfur [10], [11], [12]. Based on a reactive transport model, a previous study argued that FeS_2_ could be formed in low-sulfate Archean oceans by producing sulfide through mineralization of reduced organic sulfur or reduction of organic sulfite [12]. It is unknown whether and to what extent the organic sulfur-sourced inorganic sulfur is involved in sulfur and iron cycling, particularly in FeS_2_ formation in real natural environments. Here, we studied FeS_2_ formation in the surface sediment of Baiyangdian, a large shallow low-sulfate (∼0.16 mM) freshwater lake in north China, by analyzing a sediment core recovered in October 2019 in high depth resolution. Benefiting from the large biomass accumulation during the wet season and the undisturbed sedimentation in the dry season because of low precipitation, the lake sediment serves as an ideal laboratory for unveiling the mechanisms for FeS_2_ formation coupled to complex organic matter mineralization and iron(III) (oxyhydr)oxide reduction.

## 2 Experimental section

### 2.1 Sample collection and processing

The surface sediment core was taken from the middle of Baiyangdian Lake (115.99°N, 38.94°E, Baoding, Hebei Province, China) (Fig. S1) using a messenger-adapted gravity corer in October 2019. The sediment core was sealed immediately and transported back to laboratory on the same day in ice-cold conditions. It was then sectioned into 1 cm-thick slices and transferred into 50 mL centrifuge tubes in a glove box. Porewaters were separated from solid sediments by centrifugation at 4193 g for 0.5 h at 4 °C. Afterwards, different sulfur and iron species and organic carbon in the porewaters and solid sediments were analyzed according to the following methods.

### 2.2 Incubation experiment

*Ceratophyllum demersum* L., a grass collected from Baiyangdian, and *Spirulina* (Guguan, China) were used as the two biomass sources in incubation experiments. Under anoxic conditions in a glove box, 6 g of biomass residue and 0.3 g of hematite (99.8%, J&K Chemical Ltd., USA) were added in 50 mL sterile serum bottles, and then 10 mL of deionized water was added to completely immerse the solid sample, followed by inoculation of microorganisms with trace amount of sediment at 1 cm depth from Baiyangdian. All the bottles were sealed with butyl stoppers and aluminum crimp caps and put in the dark at room temperature. A subset of bottles was sacrificed and sampled at desired time intervals for analysis of different sulfur and iron species. Another set of incubation experiments was carried out in the same conditions but without adding hematite. The experiments were run in duplicate.

### 2.3 Analytical methods

Concentrations of sulfide in porewaters were quantified by the methylene blue method on a UV–vis spectrometer (UV2600, Shimadzu, Japan) at a wavelength of 665 nm after reacting with *N,N*-dimethyl-p-phenylenediamine (98.5%, J&K Chemical Ltd.) [13]. Concentrations of sulfate in porewaters were measured using an ion chromatograph (ThermoFisher, USA) equipped with an AS11 analytical column (ThermoFisher). Concentrations of polysulfide species, including disulfide (HS_2_^−^) and trisulfide (HS_3_^−^) in porewaters were analyzed using a derivatized method by methylating the polysulfide species with methyliodide (CH_3_I) (99.5%, Energy Chemical, China) (100 µL CH_3_I in 1 mL porewater) at 60 °C bath for 1 h to form dimethyl disulfide and dimethyl trisulfide, which were then measured by gas chromatography mass spectrometry (GC–MS, ThermoFisher) equipped with a TR-5MS column (30 × 0.25 mm i.d. × 0.25 µm) (ThermoFisher) [14]. A previous study [15] reported that sediments also contain tetrasulfide (HS_4_^−^), pentasulfide (HS_5_^−^), hexasulfide (HS_6_^−^) and heptasulfide (HS_7_^−^), which, however, were not detected by the method used in this study. Nonetheless, the measured total concentration of polysulfides should be considered as a lower bound. Concentrations of Fe^2+^ in porewaters were determined by the ferrozine method on a UV–vis spectrometer (UV2600, Shimadzu, Japan) at a wavelength of 562 nm [16].

Analysis of solid sulfur species in sediments were conducted according to the methods of previous studies [17], [18], [19], [20], [21]. For analysis of solid phase sulfur species, the sediment sample after centrifugation was firstly extracted with pure menthol (1:5, w:v) in a glove box to obtain elemental sulfur (S^0^). The concentration of S^0^ in the extract was quantified using a high-performance liquid chromatography (HPLC, Agilent 1260, USA) equipped with an Eclipse XDB-C18 column (4.6 × 150 mm) (Agilent) and a VWD detector operated at 265 nm with a mobile phase of methanol and a flow rate at 0.4 mL/min [17]. Subsequently, the solid residue was immediately added into a reactor designed by Kallmeyer et al. [18] to extract acid volatile sulfide (AVS, mainly FeS) and chromium-reducible sulfide (CRS, mainly FeS_2_) with a two-step extraction. FeS was reacted with excessive 6 N HCl to release H_2_S, and then 1 M acidic chromium dichloride solution and *N,N*-dimethylformamide were used to reduce FeS_2_ to H_2_S [18]. For both cases, the evolving H_2_S gas was trapped in a 0.5 M zinc acetate solution and measured colorimetrically through the methylene blue method [13]. Finally, the residual sample after removal of iron sulfides was freeze-dried for extracting the organic sulfur. A half of the residual sample was used to quantify the total organic sulfur by measuring the sulfate formed after combustion of the sample in Eschka's mixture (MgO:Na_2_CO_3_ = 2:1, w:w) [19]. The other half of the residual sample was added into a reducing agent comprising hydriodic acid, formic acid and red phosphorus (100:75:15, v:v:w) for reducing the R-O-SO_3_ to H_2_S, which was determined by the methylene blue method according to Johnson and Nishita [20]. The remaining organic sulfur (mainly R-SH) was quantified by subtracting R-O-SO_3_ from the total organic sulfur [21].

Sequential extractions of reactive solid iron species in sediments, including exchangeable Fe, carbonate Fe, easily reducible Fe oxides, reducible Fe oxides and magnetite were performed following the method of Poulton and Canfield [22] (detailed analytical methods can be found in Text S1). The non-reactive solid iron species (e.g., sheet silicate Fe) was not quantified. Contents of particulate organic matter (POM) in solid sediment samples were determined using a total organic carbon analyzer (TOC-L CPN, CN2600, Shimadzu). Concentration of volatile fatty acids (VFAs) in porewaters were measured using an HPLC (detailed analytical methods can be found in Text S1).

The stable carbon isotope composition (δ^13^C) of POM in the sediment was determined by a combustion elemental analyzer coupled to an isotope ratio mass spectrometry (ThermoFisher). Prior to the analysis, all samples were treated with 1 M HCl to remove inorganic carbonates. Two international standards, USGS40 and USGS62, were used to calibrate all the measurements, and the analytical precision for δ^13^C at the one standard error (1σ) level was ∼0.15‰. For stable sulfur isotope composition (δ^34^S) analyses, sulfide, FeS and FeS_2_ were transformed to Ag_2_S following their extraction, and sulfate in porewaters and sulfate from combustion of organic sulfur were precipitated to BaSO_4_ [23], [24]. The δ^34^S fractionation associated with these processes is negligible [23], [24]. The δ^34^S analysis was performed by using an elemental analyzer coupled to an isotope ratio mass spectrometer (GV Instruments, UK) with a continuous-flow method. Two standard reference materials, NBS 127 and IAEA-SO-6, were used to calibrate all the measurements, and the 1σ of the measurements was less than 0.3‰. According to Eq. 1, δ^13^C and δ^34^S values are expressed in standard as permil (‰) comparing to the Vienna Canyon Diablo Troilite (V-CDT).

(1) $$\delta^{13}C\;\text{or}\;\delta^{34}S=\left(R_{\text{sample}}/R_{\text{STD}}\right)-1$$

where *R* represents the isotope ratio of δ^13^C/δ^12^C or ^34^S/^32^S. A standard sample at fixed intervals was measured to ensure the accuracy of the stable isotope analysis [24]. The δ^34^S of FeS from 14 to 21 cm could not be measured because of insufficient amounts.

Ultrahigh resolution mass spectrometry analysis of dissolved organic matter (DOM) in the porewaters was performed using a Bruker Apex Ultra Fourier transform ion cyclotron resonance mass spectrometry (FT-ICR MS) (Germany) equipped with a 9.4 T superconducting magnet interfaced with negative-ion mode electrospray ionization. Before the analysis, the sample was extracted using the method of solid phase extraction as described previously by Dittmar et al. [25]. Samples were injected with a rate of 180 µL/h into the electrospray source, and the voltages of the capillary and spray shield were 4 and 3.5 kV, respectively. Spectra were collected over 128 scans, with an ion accumulation time of 0.6 s, a flight time of 0.0012 s and a range of 150−1200 *m/z* [26]. The datasheets that had molecular ion peaks with the ratio of signal to noisy (S/N) greater than 4 were exported to the Formula Calculator software for calculating the molecular formulas. C_0−100_H_0−200_N_0−4_O_0−30_S_0−2_ with the errors of measured mass less than 1 was used as the stringent criteria for elemental combinations of molecules. Van Krevelen (VK) diagrams were drawn according to the H/C ratio and O/C ratio of DOM molecules [27], and the identified DOM molecules were classified into 7 regions for representing 7 classes of compounds (unsaturated hydrocarbons, lignin, lipids, carbohydrates, proteins, tannins and condensed aromatics) according the boundary determined by the ratio of H/C and O/C [28].

Sulfur K-edge X-ray absorption near edge spectroscopy (XANES) analysis was performed at the Beijing Synchrotron Radiation Facility, beamline 4B7A. The X-ray beam was diffracted by a double crystal Si (111) monochromator with an energy resolution (ΔE/E) of ∼1.4 × 10^−4^, and S^0^ was used as the reference to calibrate the energy at 2472 eV. The samples were stored under a N_2_ atmosphere before the analysis. Thin layers of samples on sulfur-free Kapton tape pasting on a stainless-steel holder were prepared carefully to collect the spectra through fluorescence mode in ultra-high vacuum conditions. The range of incident X-ray energy in the analysis was from 2450 eV to 2525 eV with a step size of 0.2 eV. Normalization and calibration of the XANES spectra were processed in the software package of ATHENA, and the Gaussian Curve Fitting was used to estimate each sulfur species according to the reports of literatures [10], [29].

The microbial community composition was analyzed by high-throughput sequencing of bacterial 16S rRNA genes. DNA was extracted from each sediment sample using the FastDNA SPIN Kit (MPBiomedicals) agreeing with the manufacturer's protocol. Bac338F (ACTCCTACGGGAGGCAGCAG) and Bac806R (GGACTACHVGGGTWTCTAAT) were used as the primer pair to amplify the bacterial 16S rRNA genes. Purified amplicons were sequenced on the Illumina MiSeq platform. Paired-end reads were merged by using the FLASH software and then qualityly filtered following the procedure described previously [30]. All sequences were analyzed by defining operational taxonomic units (OTU) and each OTU was defined by 97% sequence identity. The sequences acquired in this study were deposited in the Silva database.

Fluxes of sulfate within selected depth intervals of the sediment were calculated from Fick's First Law (Eq. 2).

(2) $$J=\varphi D_{S}\frac{\partial C}{\partial z}$$

where *J* is the flux of sulfate (µmol•cm^−2^•*s* ^−^^1^), φ is the porosity, which was estimated as the volumetric water content in sediments through measuring water loss at 60 °C [31], *D_S_* was calculated from the molecular diffusion coefficient (*D_0_*, 8.9 × 10^−6^ cm^2^•*s* ^−^^1^) for sulfate in water at 18 °C [32], *D_s_* = *D_0_*/*θ*^2^ [31], [32], *θ*^2^ is the tortuosity correction (*θ*^2^ = 1 − ln(*φ*^2^)) [31] and $\frac{\partial C}{\partial z}$ is the concentration gradient of sulfate.

## 3 Results and discussion

### 3.1 Synchronization of pyrite formation with particulate organic matter (POM) mineralization

**Fig. 1:**
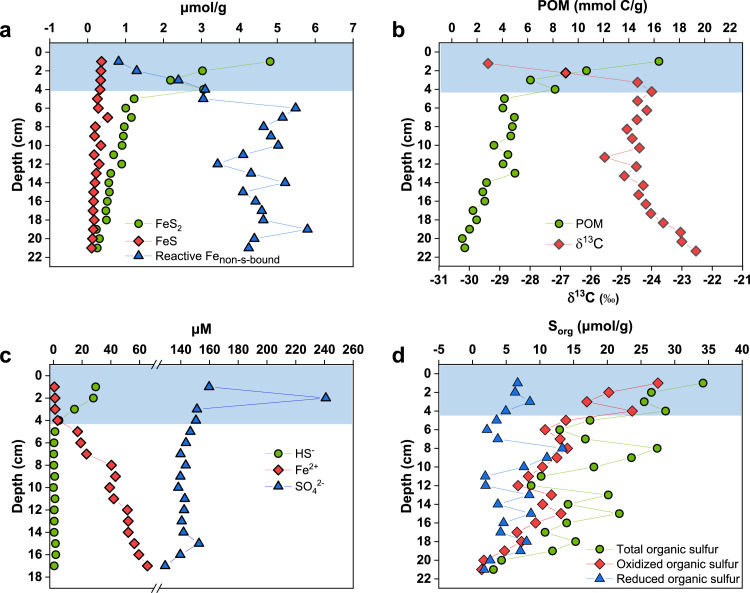
**Vertical geochemical profiles in the sediment of Lake Baiyangdian.** Concentrations of FeS_2_, FeS and non-sulfur-bound reactive iron (Reactive Fe_non-s-bound_) (a), POM along with its δ^13^C (b), HS^−^, Fe^2+^ and SO_4_^2−^ in porewaters (c) and different organic sulfur species in biomass residue (d). The active formation of FeS_2_ and HS^−^ is located within the depth interval from 1 to 4 cm, which synchronizes with that for the highest consumption of Reactive Fe_non-s-bound_ and the strongest mineralization of POM and organic sulfur.

We found that the most active zone for FeS_2_ formation is located in the top 4 cm layer. The concentration of FeS_2_ drastically decreased from the highest value of 4.81 µmol/g at 1 cm to 1.23 µmol/g at 5 cm, which was accompanied by a complementary increase of non-sulfur-bound reactive iron from 0.82 to 3.05 µmol/g and very low and nearly constant levels (∼0.3 µmol/g) of FeS (Fig. 1a). These results demonstrate that FeS_2_ is the predominant reduced iron species in the sediment. Below this depth interval, both FeS_2_ formation and non-sulfur-bound reactive iron consumption were much lower. The concentration profile of FeS_2_ was coincident with the profile of POM over the whole depth from 1 to 21 cm (Fig. 1b), suggesting that FeS_2_ formation is synchronized with POM mineralization coupled to MIR. Corresponding to the depth-dependent mineralization extent of POM, the stable carbon isotope composition (δ^13^C) of POM increased rapidly from −29.4 permil (‰) at 1 cm to −24.0 ‰ at 4 cm, remaining nearly constant (∼−24.5‰) within the 5 to 13 cm interval and then increased moderately from −24.3‰ at 14 cm to −22.5‰ at 21 cm (Fig. 1b). These results are consistent with the fact that ^12^C-einriched molecules are consumed faster than their ^13^C-einriched counterparts [33]. The strong and rapid mineralization of POM in the surface layer creates highly anoxic conditions with the redox potential (Eh) ranging from −49 mV at 1 cm to −18 mV at 4 cm (Fig. S2). Similar to FeS_2_, the concentrations of other reduced inorganic sulfur species (sulfide and polysulfides) in porewaters peaked at 1 cm depth and then rapidly decreased to low levels at 4 cm (Figs. 1c and S3). Sulfide concentrations exceeded Fe^2+^concentrations by far from 1 to 3 cm depth, whereas the trend was reversed at 5 cm and lower depths (Fig. 1c), reflecting a shift from Fe^2+^ limitation to sulfide limitation in FeS_2_ formation.

### 3.2 Production of inorganic sulfur from organic sulfur mineralization

**Fig. 2:**
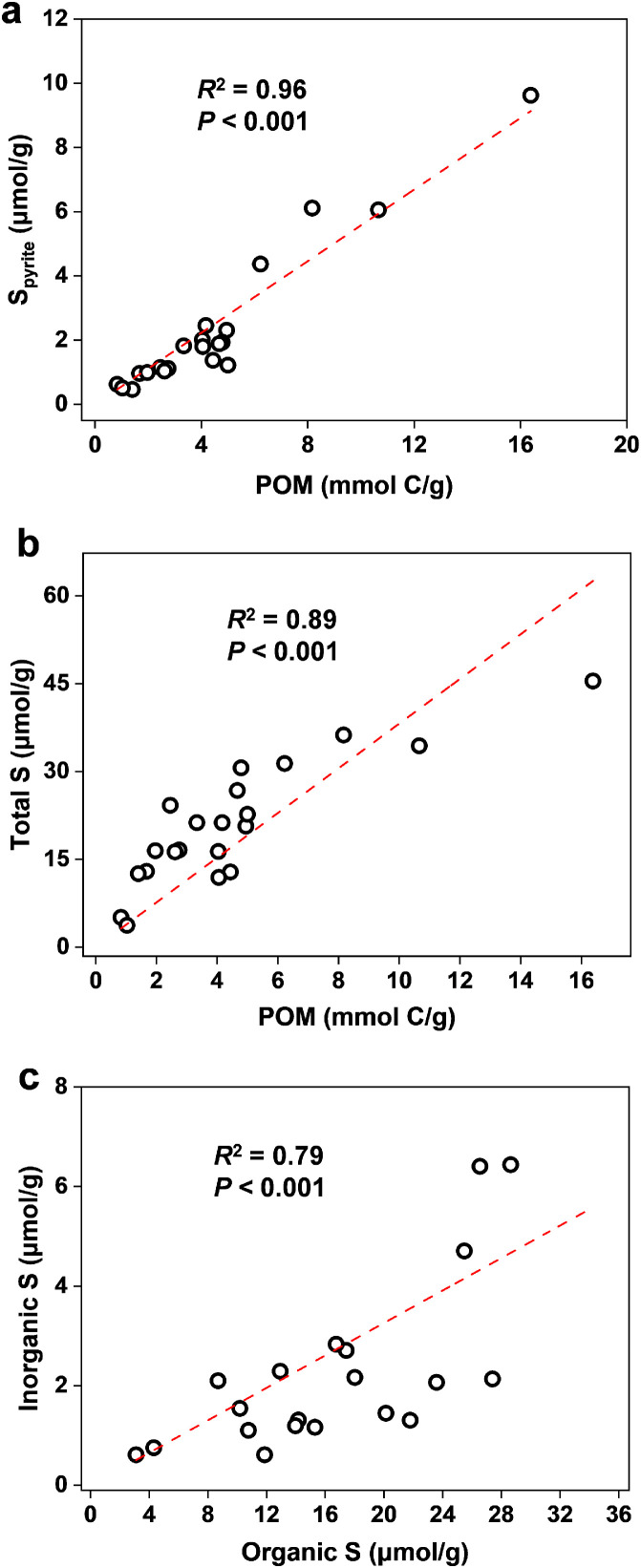
**Correlations of (a) concentrations of organic carbon in POM versus concentrations of pyrite, (b) concentrations of organic carbon in POM versus concentrations of total sulfur and (c) concentrations of organic sulfur versus concentrations of inorganic sulfur**.

In contrast to the reduced inorganic sulfur species, the concentrations of sulfate remained nearly constant over the whole depth except for a very high subsurface peak (241 µM) at 2 cm (Fig. 1c), which indicates its origin from organic sulfur in the sedimentary biomass [11]. Most (∼76%) of the sulfate produced at 2 cm depth diffuses upwards to the above layer and then to the overlying water (containing ∼160 µM sulfate as measured) resulting from the much higher diffusion constant within the sediment at 1 cm because of the larger porosity as compared to the sediments at 2 cm and 3 cm (Table S1). In synchronization with the content profile of POM, the total organic sulfur content and the content of oxidized organic sulfur in POM decreased with depth, and the largest loss gradient was shown from 1 to 4 cm depth (Fig. 1d). According to the FT-ICR MS analysis of porewater DOM, the organic sulfur in proteins, lipids and carbohydrates in POM is rapidly mineralized and released as dissolved organic sulfur in the top layer (Fig. S4). The content of reduced organic sulfur calculated by the sulfur mass balance between these two organic sulfur species was relatively constant over the whole depth (Fig. 1d), implying rapid mineralization. The predominant role of organic sulfur in producing inorganic sulfur, including sulfate and that in FeS_2_ can be further seen from the following three positive correlation relationships: FeS_2_ concentration and POM content (*R*^2^ = 0.96, *P* < 0.001); total sulfur content (the sum of all measured inorganic sulfur content, including FeS_2_, FeS, S^0^, sulfide, polysulfides, and sulfate and organic sulfur in POM) and POM content (*R*^2^ = 0.89, *P* < 0.001); inorganic sulfur content and organic sulfur content in POM (*R*^2^ = 0.79, *P* < 0.001) (Fig. 2).

### 3.3 Multiple evidences for sulfide and pyrite formation from reduced organic sulfur

**Fig. 3:**
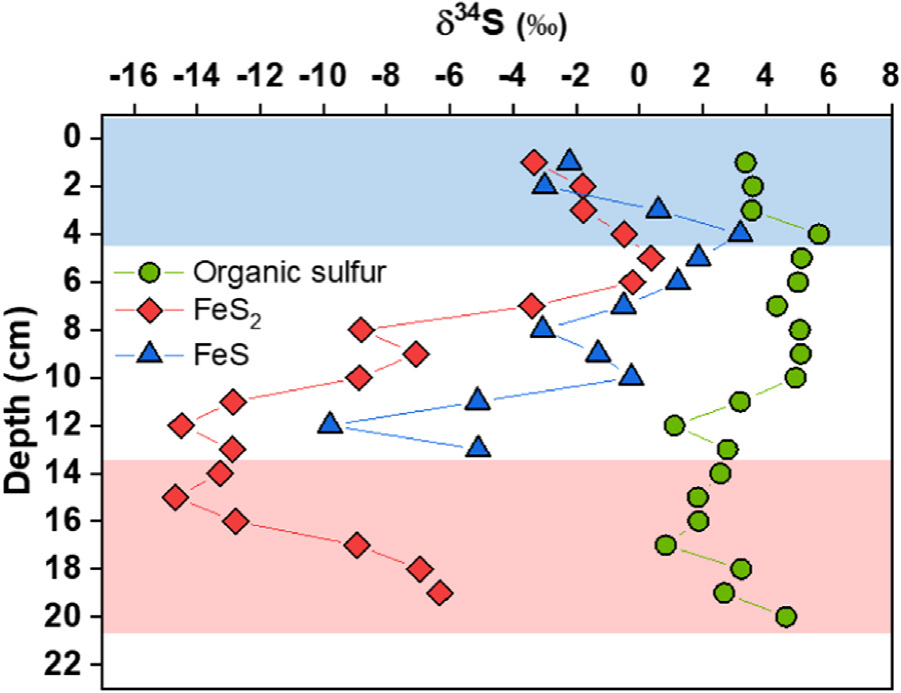
**δ^34^S of FeS_2_, FeS and organic sulfur throughout the sediment core of Lake Baiyangdian.** Three different zones with contrasting δ^34^S patterns are identified in correspondence to mineralization intensity of POM.

Based on our data, we conclude that the majority of sulfide involved in FeS_2_ formation in the top 4 cm layer is mainly produced by mineralization of reduced organic sulfur in POM rather than from sulfate derived from oxidized organic sulfur via the MSR pathway. First, the concentration of sulfate remained nearly constant at most examined depths, which is indicative of minimal consumption of sulfate by MSR. Second, the peak concentration of sulfide appeared at 1 cm depth and was 1 cm above the peak concentration of sulfate, confirming its earlier appearance. Third, the reduction ratio (59%) of sulfate (160 µM) by MSR to reduced inorganic sulfur (94 µM through summing all the sulfur in measured sulfide and polysulfides) in the porewater at 1 cm depth would be unrealistically high assuming steady-state reaction conditions. MSR is reported to play an important role in organic mineralization in freshwater sediments with external sulfate supplied by overlying water, which is characterized by decline of sulfate concentration with increasing sediment depth [34], [35]. However, herein the endogenous origin of sulfate from MIR-driven organic mineralization greatly inhibits the MSR pathway. A strong piece of evidence for sulfide production from reduced organic sulfur comes from the analysis of stable sulfur isotope compositions (δ^34^S) of FeS_2_, FeS and organic sulfur in POM (Fig. 3 and Table S2). The slightly depleted δ^34^S of FeS_2_ (−3.3‰ to −0.5‰) and FeS (−2.2‰ to 3.9‰) relative to organic sulfur (3.4‰ to 5.7‰) in the top 4 cm layer indicates the same source of sulfur. In anoxic conditions (Eh less than −18 mV), the precipitation by Fe^2+^produced by MIR and the abiotic oxidation by iron(III) (oxyhydr)oxides are the two major sinks for sulfide in the absence of other primary oxidants or precipitating metal ions. These two reactions cause negligible changes in δ^34^S of sulfide [36], [37]. In addition, the MSR pathway can be ruled out by the very close δ^34^S values between sulfate (9.4‰) and sulfide (6.6‰) at 1 cm depth. If MSR were the dominant pathway for sulfide production, more depleted δ^34^S (generally around 30‰ or higher) in natural environments would be shown for sulfide relative to sulfate [38], [39], [40]. In fact, the sulfate-reducers identified in the sediment are dominated by complete oxidizing sulfate-reducing microbes (79% to 99%) (Table S3), which use sulfate to completely oxidize organic substrates into carbon dioxide and cause even larger sulfur isotope fractionations (up to 42‰) [41]. With increasing depth from 5 to 13 cm, the contribution of the MSR pathway to sulfide production becomes relatively more important because of the limited mineralization of POM (as reflected by the nearly constant content and δ^13^C of POM, Fig. 1b), which is evidenced by the more depleted δ^34^S (up to −14.7‰) of FeS_2_ and FeS (Fig. 3). Nonetheless, the FeS and FeS_2_ formed within this depth interval were much less than those in the top 4 cm layer. As the depth increases further from 14 to 21 cm, sulfide production gradually shifts back to mineralization of reduced organic sulfur as indicated by the enriched δ^34^S (−12.8‰ to −6.6‰) of FeS_2_ and FeS. This is supported by the moderately enhanced POM mineralization within this depth interval as shown by the decreased content and enriched δ^13^C of POM with increasing depth as well as the accumulation of fermentation-derived acetic acid and propanoic acid with increasing depth (Fig. S5).

### 3.4 Reduced organic sulfur-driven pyrite formation in biomass incubation

**Fig. 4:**
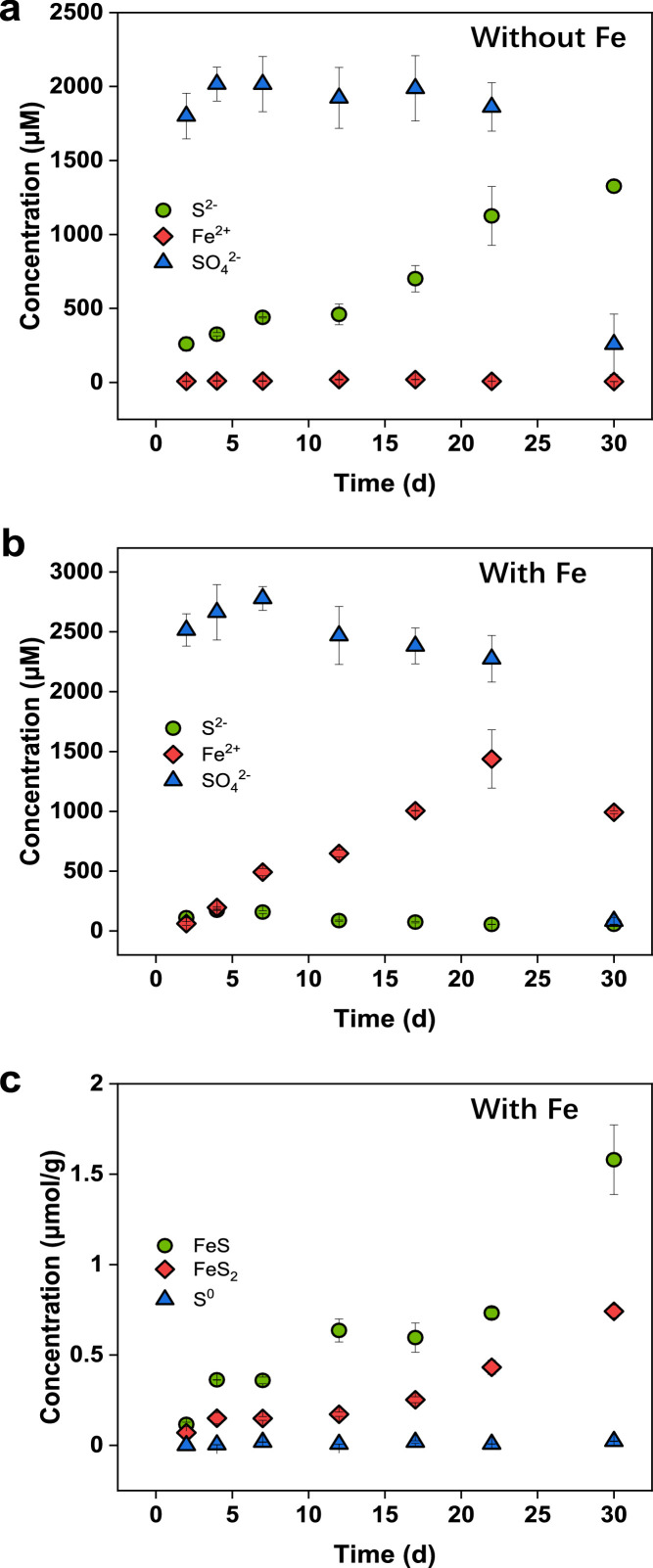
**Concentrations of different iron species and sulfur species during the incubation of *Ceratophyllum demersum* L.** FeS and FeS_2_ were rapidly formed at 2 d in the presence of hematite. With the exception of *Ceratophyllum demersum* L. at 30 d in the presence or absence of hematite, the concentrations of sulfate kept relatively constant, indicating low activity of MSR pathway.

**Fig. 5:**
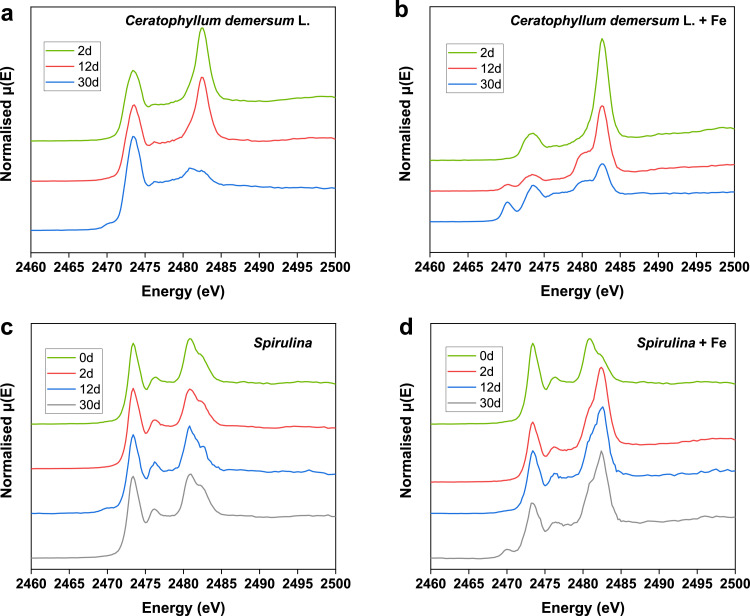
**Relative absorbance of normalized S K-edge XANES spectra of biomass samples from incubation experiments.** (a) *Ceratophyllum demersum* L. without hematite. (b) *Ceratophyllum demersum* L. with hematite. (c) *Spirulina* without hematite. (d) *Spirulina* with hematite. The presence of hematite greatly accelerated the mineralization of organic sulfur, especially reduced organic sulfur in the biomass residue.

FeS_2_ and FeS were readily formed in less than 2 d in anoxic incubation of a lake grass (*Ceratophyllum demersum* L.) collected from Baiyangdian or cynobacteria (*Spirulina*) with hematite and inoculation of sediment in the absence of external sulfate (Figs. 4 and S6). The presence of hematite resulted in much more formation of inorganic sulfur, especially reduced inorganic sulfur (including sulfide and that in FeS_2_ and FeS) compared to the absence of hematite. In the presence of hematite, sulfide produced from the mineralization of biomass was rapidly consumed by the reaction with hematite to form iron sulfides, which in turn promoted the decomposition of biomass to generate more reduced inorganic sulfur. With the exception of *Ceratophyllum demersum* L. at the end of incubation (30 d), the sulfate released by the two biomasses kept nearly constant regardless of the presence of hematite, indicating the negligibility of the MSR pathway during most of the period. Consistently, the δ^34^S of sulfate remained stable and was nearly identical to the δ^34^S of organic sulfur in *Ceratophyllum demersum* L. until 30 d with an abrupt δ^34^S enrichment (∼6.4‰) signifying the activation of MSR (Table 1). As reaffirmed by the close δ^34^S values between FeS_2_, FeS and organic sulfur in biomass (Tables 1 and S4), the sulfide involved in FeS_2_ formation originated from mineralization of reduced organic sulfur rather than from biomass-derived sulfate via the MSR pathway. X-ray absorption near-edge structure (XANES) spectroscopy analysis of biomass samples from incubation confirmed that in the presence of hematite, the reduced organic sulfur was rapidly mineralized in less than 2 d, and the mineralization of reduced organic sulfur was much faster than that of oxidized organic sulfur (Fig. 5; Tables S5 and S6).

**Table 1:** **Stable sulfur isotope compositions (δ**^**34**^**S) of different sulfur species during the incubation of*****Ceratophyllum demersum***L.*

Day	Without Fe				With Fe			
	δ^34^S [HS^−^] (‰)	δ^34^S [SO_4_^2−^] (‰)	δ^34^S [S*_org_*] (‰)		δ^34^S [F*e*S] (‰)	δ^34^S [F*e*S_2_] (‰)	δ^34^S [SO_4_^2−^] (‰)	δ^34^S [S*_org_*] (‰)
2	4.1	4.5	4.6		4.5	2.1	4.1	5.1
4	3.6	4.4	4.8		3.9	1.2	3.9	4.1
7	6.2	4.6	4.9		5.4	1.9	4.2	5.8
12	7.0	5.4	6.4		5.5	2.3	4.7	5.7
17	6.3	4.4	4.8		5.7	3.0	4.5	4.5
22	5.8	5.7	6.0		4.8	1.0	4.6	5.2
30	4.4	11.2	5.2		6.0	4.6	10.8	4.9

⁎ Without the presence of hematite, the δ^34^S values of sulfide, sulfate and organic sulfur in biomass remained nearly constant and were close to each other during the whole incubation with the exception of the enriched δ^34^S of sulfate at 30 d. A similar trend was observed for the δ^34^S values of sulfate, FeS and organic sulfur in biomass in the presence of hematite. FeS_2_ had a slight depletion of δ^34^S by about 2.5‰ relative to FeS. For both cases of with and without the presence of hematite, the δ^34^S of sulfate at 30 d showed an enrichment by about 6.4‰ relative to other times, suggesting the initiation of microbial sulfate reduction (MSR) coupled to biomass mineralization at 30 d.

## 4 Conclusion

We show unambiguously that mineralization of reduced organic sulfur in biomass can release abundant sulfide to effectively drive FeS_2_ formation in low-sulfate freshwater sediments. The instant and peak release of sulfide from organic matter mineralization is expected to play an important role in FeS_2_ formation in many low-sulfate environments, which also sheds light on sulfur cycling and FeS_2_ formation in sea sediments of the early Earth where sulfate concentrations are typically less than 10 µM [42], [43]. Another case of concern is that when accidental input of large amounts of organic matter by decayed algae bloom occurs, the reduced organic sulfur-driven formation of sulfide and FeS_2_ may also greatly affect the biogeochemical cycling of toxic trace metals that have high affinities for sulfide or have been previously adsorbed/precipitated at the surface of iron(III) (oxyhydr)oxides involved in POM mineralization.

## Declaration of competing interest

The authors declare that they have no conflicts of interest in this work.

## Appendix Supplementary materials

Image, application 1
