# Supplementary material for: A new pathway for pyrite formation in low-sulfate sediments driven by mineralization of reduced organic sulfur
Source: Fundam Res. 2023 Sep 14;5(4):1607–13. doi: 10.1016/j.fmre.2023.08.003 (PMC12327826; doi:10.1016/j.fmre.2023.08.003)
Supplement: Supplementary file 1 [file mmc1.docx]

SUPPLEMENTARY INFORMATION

A New Pathway for Pyrite Formation in Low-Sulfate Sediments Driven by Mineralization of Reduced Organic Sulfur

Chenhui Wei, Shujun Yin, Andreas Kappler, Shu Tao, Dongqiang Zhu^*^

Correspondence to: zhud@pku.edu.cn

This supplementary file includes:

Text S1;

Fig. S1 to Fig. S6;

Table S1 to Table S6;

References.

**Text S1**

**Steps of reactive solid iron species sequential extractions.** (a) Exchangeable Fe (Fe_exc_) was extracted by 1 M magnesium chloride solution; (b) carbonate Fe (Fe_carb_) was extracted by 1 M sodium acetate solution; (c) easily reducible Fe oxides (Fe_ox1_) were extracted by 1 M hydroxylamine hydrochloride solution; (d) reducible Fe oxides (Fe_ox2_) were extracted by 50 g/L sodium dithionite solution; (e) magnetite (Fe_mag_) was extracted by 0.2 M ammonium oxalate/0.17 M oxalic acid solution. The concentration of iron extracted from each of the five leachates above was measured by the ferrozine method after reducing all Fe^3+^ to Fe^2+^ by hydroxylamine hydrochloride. Because acetate can quantitatively solubilize FeS (step a), the Fe_carb_ content of sediments was measured as the difference between the acetate and FeS extractions [1]. Non-sulfur-bound reactive iron (Reactive Fe_non-s-bound_) in the sediment was quantified as the sum of Fe_exc_, Fe_carb_, Fe_ox1_, Fe_ox2_ and Fe_mag_ [1,2].

**Analysis method of VFAs.** Concentration of VFAs in porewaters were measured using an HPLC with a VWD detector at a wavelength of 210 nm A 4.6 × 250 mm Eclipse Plus Phenyl-Hexyl column (Agilent) was used to separate the VFAs, and NaH_2_PO_4_ (20 mM) and CH_3_CN (95:5, v:v) at pH 2 was used as the mobile phase with a flow rate of 1 mL/min.

**Supplementary Figures**

**Fig. S1.** Map showing the Baiyangdian Lake location and sampling site in the lake (red dot). (a) map of China; (b) map of Hebei Province; (c) map of Baiyangdian Lake. Figures (a) and (b) are from the maps with the audit numbers of GS(2020)4619 and GS(2019)3333, respectively.

**Fig. S2.** Depth profile of Eh in the sediment of Baiyangdian Lake.

**Fig. S3.** Depth profiles for concentrations of polysulfides in porewaters. Trisulfide (HS_3_^-^) and bisulfide (HS_2_^-^) were the major polysulfide species identified, and both of them were mainly formed in the top 4 cm layer.

**Fig. S4.** Van Krevelen diagrams for formulas of sulfur containing molecules assigned via FT-ICR MS analysis of DOM in porewaters within a depth interval of (**a**) 1 to 4 cm, (**b**) 5 to 13 cm and (**c**) 14 to 19 cm. The areas in the diagrams show the different classes of biochemical compounds. (**d**) Intensity-weighted relative abundances of different classes of compounds for the sulfur containing molecules. Compared with those within the depth intervals of 5 to 13 cm and 14 to 19 cm, the sulfur containing molecules within the depth interval of 1 to 4 cm had higher ratios of proteins and lipids.

**Fig. S5.** Depth profiles of concentrations of acetic acid and propionic acid in porewaters. Concentrations of acetic acid remained nearly constant (15 μM on average) from 1 to 13 cm and then increased rapidly from 29.4 μM at 14 cm to 90.5 μM at 19 cm. Propionic acid was only detected at depths of 14 cm and lower, and the concentrations increased rapidly from 8.1 μM at 14 cm to 49.5 μM at 19 cm.

**Fig. S6.** Concentrations of different iron species and sulfur species during the incubation of *Spirulina*. FeS and FeS_2_ were rapidly formed at 2 d in the presence of hematite.

**Supplementary Tables**

**Table S1.** Calculation of diffusive sulfate fluxes within the top layers of sediment.

| **Depth**  **(cm)** | **Porosity** | **Tortuosity correction** | **Diffusion coefficient**  **(cm^2^•s^-1^)** | **Flux**  **(μmol•cm^-2^•s^-1^)** |
| --- | --- | --- | --- | --- |
| 1 | 0.72 | 1.65 | 5.38 × 10^-6^ | 3.15 × 10^-7*^ |
| 2 | 0.43 | 2.67 | 3.33 × 10^-6^ |  |
| 3 | 0.37 | 2.99 | 2.98 × 10^-6^ | 9.89 × 10^-8†^ |

^*^Diffusion direction is from 2 cm to 1 cm. ^†^Diffusion direction is from 2 cm to 3 cm.

**Table S2.** δ^34^S of FeS_2_, FeS and organic sulfur throughout the sediment core of Lake Baiyangdian.

| **Depth**  **(cm)** | **δ^34^S_[FeS]_**  **(‰)** | **δ^34^S_[FeS2]_**  **(‰)** | **δ^34^S_[Sorg]_**  **(‰)** |
| --- | --- | --- | --- |
| 1 | -2.2 | -3.3 | 3.4 |
| 2 | -3.7 | -1.8 | 3.6 |
| 3 | 0.6 | -1.8 | 3.6 |
| 4 | 3.2 | -0.5 | 5.7 |
| 5 | 1.9 | 0.4 | 5.1 |
| 6 | 1.2 | -0.2 | 5.0 |
| 7 | -0.5 | -3.4 | 4.2 |
| 8 | -3.1 | -8.8 | 6.0 |
| 9 | -1.3 | -7.1 | 5.1 |
| 10 | -0.3 | -8.9 | 5.0 |
| 11 | -5.1 | -12.9 | 3.2 |
| 12 | -9.8 | -14.5 | 1.1 |
| 13 | -5.1 | -12.9 | 2.8 |
| 14 |  | -13.3 | 2.6 |
| 15 |  | -14.7 | 1.9 |
| 16 |  | -12.8 | 1.9 |
| 17 |  | -9.0 | 0.8 |
| 18 |  | -7.0 | 3.2 |
| 19 |  | -6.3 | 2.7 |
| 20 |  |  | 4.6 |

**Table S3.** Abundances (%) of sulfate reducing bacteria (SRB) and complete-oxidizing SRB at the genus level in the sediment of Baiyangdian at varying depths according to 16S rRNA analysis of microbial community diversity, along with percentages (%) of complete-oxidizing SRB to the total SRB.

| **SRB** | **Oxidation of substrate** | **Abundances (%)** | | | | | | | |
| --- | --- | --- | --- | --- | --- | --- | --- | --- | --- |
|  |  | **1 cm** | **2 cm** | **5 cm** | **9 cm** | **13 cm** | **17 cm** | **21 cm** |  |
| Desulfatirhabdium | C^*^ | 0.266 | 0.065 | 0.116 | 0.031 | 0.004 | 0 | 0 |  |
| Desulfococcus | C | 0.015 | 0.012 | 0.004 | 0. | 0 | 0 | 0 |  |
| Desulfovermiculus | C | 0.031 | 0.008 | 0.019 | 0.004 | 0 | 0 | 0 |  |
| Desulfomonile | C | 0.069 | 0.050 | 0.058 | 0.027 | 0.004 | 0.004 | 0 |  |
| Desulfovirga | C | 0.089 | 0.035 | 0.027 | 0 | 0.008 | 0 | 0 |  |
| Desulfobacterium_  catecholicum_group | C | 0.820 | 0.158 | 0.316 | 0.039 | 0.004 | 0.008 | 0.023 |  |
| Desulfatitalea | C | 0.031 | 0 | 0.008 | 0 | 0 | 0 | 0.008 |  |
| Desulfobacca | C | 0.073 | 0.104 | 0.089 | 0.031 | 0.181 | 0.1 | 0.123 |  |
| Desulfonema | C | 0 | 0 | 0 | 0 | 0 | 0 | 0.004 |  |
| Desulfatibacillum | C | 0.015 | 0 | 0 | 0 | 0 | 0 | 0 |  |
| Desulfobacca | C | 0.331 | 0.031 | 0 | 0.008 | 0 | 0.004 | 0 |  |
| Desulfobacterium | C | 0.035 | 0.023 | 0 | 0 | 0 | 0 | 0 |  |
| Desulfatiglans | C | 0.366 | 0.347 | 0.466 | 0.081 | 0.089 | 0.065 | 0.05 |  |
| Desulfatiferula | I ^†^ | 0.023 | 0.004 | 0 | 0 | 0 | 0 | 0 |  |
| Desulfosporosinus | I | 0.015 | 0 | 0.035 | 0 | 0 | 0 | 0 |  |
| Desulfovibrio | I | 0.1 | 0.015 | 0.027 | 0.004 | 0 | 0 | 0.012 |  |
| Desulforhopalus | I | 0.077 | 0.015 | 0.008 | 0.008 | 0 | 0 | 0 |  |
| Desulfobulbus | I | 0.173 | 0.069 | 0.042 | 0.012 | 0.004 | 0.004 | 0.031 |  |
| Desulfocapsa | I | 0.015 | 0 | 0 | 0 | 0 | 0 | 0 |  |
| Desulfomicrobium | I | 0.069 | 0.023 | 0.008 | 0.008 | 0 | 0.004 | 0.012 |  |
| **Total abundances (%)** | | 2.6 | 0.96 | 1.2 | 0.25 | 0.29 | 0.19 | 0.26 |  |
| **Percentages of complete-oxidizing SRB (%)** | | 82 | 87 | 90 | 88 | 99 | 96 | 79 |  |

^*^Complete oxidation. ^†^Incomplete oxidation. Classification of SRB was performed according to references [3,4].

**Table S4.** Stable sulfur isotope compositions (δ^34^S) of different sulfur species during the incubation of *Spirulina*. Without the presence of hematite, sulfide had slightly depleted **δ**^34^S (1.5 to 3.9‰) compared to organic sulfur in biomass. Similarly, the **δ**^34^S of FeS and FeS_2_ was only slightly lower than that of organic sulfur in biomass (up to 2.9‰ and 4.4‰, respectively) with the presence of hematite. The δ^34^S of sulfate was not measured because of insufficient amounts.

|  | **Without Fe** | |  | **With Fe** | | |
| --- | --- | --- | --- | --- | --- | --- |
| **Day** | **δ^34^S_[HS_^-^_]_**  **(‰)** | **δ^34^S_[Sorg]_**  **(‰)** |  | **δ^34^S_[FeS]_**  **(‰)** | **δ^34^S_[FeS2]_**  **(‰)** | **δ^34^S_[Sorg]_**  **(‰)** |
| 2 | 4.2 | 6.0 |  | 4.8 | 2.6 | 6.2 |
| 4 | 3 | 6.5 |  | 7.2 | 5.2 | 6.7 |
| 7 | 3.5 | 6.3 |  | 5.7 | 2.4 | 6.8 |
| 12 | 2.9 | 6.8 |  | 3.9 | 3.7 | 6.2 |
| 17 | 2.4 | 6.3 |  | 5.3 | 2.8 | 6.4 |
| 22 | 5.2 | 6.7 |  | 5.8 | 2.3 | 6.6 |
| 30 | 4.2 | 6.0 |  | 3.4 | 3.5 | 6.3 |

**Table S5.** Atomic fractions (%) of different sulfur species during the incubation of *Ceratophyllum demersum* L. as determined by the Gaussian Curve Fitting method in S K-edge XANES spectra. The biomass contained R-SH, R-SS-R, R-SO_3_ and R-O-SO_3_. In the presence of hematite, the relative abundance (%) of R-SH was much lower than that without the presence of hematite, and a reversed trend was shown for R-O-SO_3_. FeS_2_ and FeS were gradually formed in the presence of hematite.

|  | **FeS**  **(%)** | **FeS_2_**  **(%)** | **R-SH**  **(%)** | **R-SS-R (%)** | **R-SO**  **(%)** | **R-SO_2_**  **(%)** | **R-SO_3_**  **(%)** | **R-O-SO_3_ (%)** |
| --- | --- | --- | --- | --- | --- | --- | --- | --- |
| **2 d**  **without Fe** | 0 | 0 | 77.2 | 2.5 | 0 | 0 | 1.7 | 18.7 |
| **12 d**  **without Fe** | 0 | 0 | 77.9 | 1.8 | 0 | 0 | 5.7 | 14.6 |
| **30 d**  **without Fe** | 4.2 | 0 | 82.5 | 0 | 4.5 | 2.4 | 4.1 | 2.4 |
| **2 d**  **with Fe** | 0 | 0 | 41.7 | 11.7 | 0 | 0 | 11.2 | 35.3 |
| **12 d**  **with Fe** | 8.1 | 4.7 | 36.3 | 0 | 0 | 5.1 | 19.3 | 26.6 |
| **30 d**  **with Fe** | 18.9 | 5.2 | 40.9 | 3.3 | 10.5 | 7.2 | 5.5 | 8.6 |

**Table S6.** Atomic fractions (%) of different sulfur species during the incubation of *Spirulina* determined by the Gaussian Curve Fitting method in S K-edge XANES spectra. The biomass contained R-SH, R-SO, R-SO_2_, R-SO_3_ and R-O-SO_3_, and R-SH was the major sulfur species with the highest relative abundance (> 50%). In the absence of hematite, the relative abundances of all sulfur species kept nearly constant during the whole incubation. In the presence of hematite, the relative abundance of R-SH gradually decreased, and FeS_2_ and FeS were gradually formed.

|  | **FeS**  **(%)** | **FeS_2_**  **(%)** | **R-SH**  **(%)** | **R-SS-R (%)** | **R-SO**  **(%)** | **R-SO_2_**  **(%)** | **R-SO_3_**  **(%)** | **R-O-SO_3_ (%)** |
| --- | --- | --- | --- | --- | --- | --- | --- | --- |
| **0 d**  **without Fe** | 0 | 0 | 59.2 | 0 | 17.0 | 5.4 | 8.0 | 10.3 |
| **2 d**  **without Fe** | 0 | 0 | 56.7 | 0 | 18.8 | 5.9 | 8.3 | 10.3 |
| **12 d**  **without Fe** | 0 | 0 | 57.4 | 0 | 17.6 | 5.7 | 9.3 | 10.0 |
| **30 d**  **without Fe** | 0 | 0 | 56.9 | 0 | 18.3 | 5.5 | 8.5 | 10.8 |
| **2 d**  **with Fe** | 0 | 0 | 50.0 | 0 | 11.6 | 4.5 | 8.6 | 25.3 |
| **12 d**  **with Fe** | 4.0 | 0 | 50.0 | 0 | 13.7 | 4.7 | 7.9 | 19.8 |
| **30 d**  **with Fe** | 6.5 | 8.2 | 43.0 | 0 | 10.1 | 4.4 | 7.5 | 20.3 |

**References**

1. S.W. Poulton, D.E. Canfield, Development of a sequential extraction procedure for iron: implications for iron partitioning in continentally derived particulates. Chem. Geol. 214 (2005) 209–221.
2. J. Liu, A. Pellerin, G. Antler, et al., Early diagenesis of iron and sulfur in Bornholm Basin sediments: The role of near-surface pyrite formation. Geochim. Cosmochim. Acta 284 (2020) 43–60.
3. J. Detmers, V. Bruchert, K. S. Habicht, et al, Diversity of sulfur isotope fractionations by sulfate-reducing prokaryotes. Appl. Environ. Microbiol. 67 (2001) 888–894.
4. W. B. Whitman, *Bergey's manual of systematics of archaea and bacteria*, W.B. Whitman, P. DeVos, J. Chun, S. Dedysh, B. Hedlund, P. Kämpfer, F. Rainey, M. Trujillo, Eds. (Wiley, New Jersey, 2015).
